# Special Issue “Clinical Frontiers in Percutaneous Coronary Intervention”

**DOI:** 10.3390/jcm12185969

**Published:** 2023-09-14

**Authors:** Marcel A. M. Beijk

**Affiliations:** Department of Cardiology, Amsterdam University Medical Center, University of Amsterdam, 1105 AZ Amsterdam, The Netherlands; m.a.beijk@amsterdamumc.nl; Tel.: +31-20-5669111

In the last decade, significant advancements have been made in the field of percutaneous coronary interventions (PCIs) with the development of new devices and drugs, the application of new technology and the utilization of artificial intelligence/machine learning, and new indications for revascularization. As a result, clinical outcomes after PCIs have improved dramatically; however, coronary artery disease (CAD) remains the leading cause of death in developed countries [1]. Therefore, the need for continuous progress remains. Moreover, as the life expectancy of the general population grows, patients will present with increasing high-risk comorbidities and more complex CAD. Due to this broad and heterogeneous spectrum of CAD patients, complex cases should be discussed by multidisciplinary heart teams to identify the optimal revascularization strategy for each specific patient. This Special Issue of the *Journal of Clinical Medicine* (*JCM*) aimed to collect high-quality research dedicated to clinical frontiers in percutaneous coronary intervention.

The PCI of chronic total occlusions (CTOs) remains a challenge for interventional cardiologists, although major advancements have been achieved due to the development of dedicated material and the growing expertise among CTO operators. In patients with CTO undergoing PCIs, the prediction of technical success is essential for patient selection for percutaneous revascularization and pre-procedural planning. In study by Nakachi et al., the predictability of machine learning (ML) models for technical results of CTO-PCI was investigated, and the performances were compared to the results from existing CTO scores, including J-CTO, CL, and CASTLE scoring systems [2]. Data were extracted from the Japanese CTO-PCI expert registry. A large cohort of consecutive patients (*n* = 8760) undergoing CTO-PCI was enrolled, and technical success was achieved in 91.2% of patients. The performance of prediction using five ML models (random forest; extreme gradient boosting (XGBoost); deep neural networks; support vector machine classifier; and L2-regularized logistic regression) was evaluated using the area under the receiver-operating curve (AUC). The authors found that XGBoost outperformed the conventional prediction scores (XGBoost: AUC 0.760 (95% confidence interval (CI): 0.740–0.780); J-CTO: AUC 0.697 (95%CI: 0.675–0.719); CL: AUC 0.662 (95%CI: 0.639–0.684); CASTLE: AUC 0.659 (95%CI: 0.636–0.681); *p* < 0.005 for all). In addition, the XGBoost model showed acceptable concordance between the predicted and observed probabilities of CTO-PCI failure wherein the presence of a calcified lesion was the leading predictor. This study was the first large-scale evaluation of ML used to predict the success of CTO-PCI and showed that ML algorithms represent a promising technique. ML techniques can provide accurate and distinct information regarding the likelihood of technical success in CTO-PCI and thereby may help select the optimal treatment for CTO patients.

The visual assessment of a left main coronary artery (LMCA) stenosis can be difficult due to the anatomy that may lead to misclassification in almost one-third of patients with a LMCA stenosis when compared to functional assessment with intracoronary physiology, i.e., fractional flow reserve [3]. The quantitative flow ratio (QFR) enables the assessment of the hemodynamic relevance of a coronary stenosis based solely on two angiographic projections, without the need for pressure wires or medications. Milzi et al. aimed to evaluate the association of intravascular imaging with the QFR in the context of LMCA stenosis [4]. In 53 patients with LMCA disease, the association between anatomic assessment using optimal coherence tomography (OCT) or intravascular ultrasound (IVUS) and functional assessment as determined via the QFR was tested. The minimal luminal area (MLA) of the LMCA as measured using OCT or IVUS showed a consistent correlation with the QFR (R = 0.61, *p* < 0.001). The QFR could predict a LMCA MLA < 6 mm^2^ with excellent diagnostic accuracy (AUC 0.919) and a LMCA MLA < 4.5 mm^2^ with good accuracy (AUC 0.798). The QFR is a novel technology that may aid operators in assessments and decision making regarding a LMCA stenosis.

The clinical outcomes of patients undergoing PCI for cardiogenic shock secondary to total occlusive unprotected LMCA lesion-related acute myocardial infarction (AMI) were evaluated in a retrospective analysis by Beijk et al. [5]. A total of 49 patients were included, and the majority of patients (51%) had cardiac arrest prior to or during PCI. The thirty-day mortality rate was very high (78%). Patients who survived for 30 days (*n* = 11) were followed for a median of 9.9 years (interquartile range: 4.7–13.6). The long-term all-cause mortality rate was 84% and was only independently associated with cardiac arrest prior to or during PCI (hazard ratio (HR) 2.02, 95% confidence interval 1.02–4.01, *p* = 0.043). Moreover, 30-day survivors with severe left ventricular dysfunction had poor long-term prognoses. Importantly, the clinical outcomes were dependent on the characteristics of the patients. In an overview of the literature, the authors showed that a comparison of previous studies with AMI due to unprotected LMCA displayed remarkable heterogeneity in cardiogenic shock (12 to 76%) and TIMI flow (which varied from 0 to 3) affecting 30-day mortality (6.2 to 78%).

This Special Issue of the *Journal of Clinical Medicine* (*JCM*) publishes three studies from Australia with data extracted from the GenesisCare Cardiovascular Outcomes Registry (GCOR-PCI). Established in 2008, this GCOR registry is an internally funded ongoing registry for subjects undergoing PCI at 12 Australian private hospitals. In the first study by Eccleston et al., the in-hospital and long-term outcomes after PCI with the Xience (Abbott Laboratories, Abbott Park, IL, USA) everolimus-eluting stents (EESs) were reported [6]. Including consecutive patients from January 2015 to January 2019 treated exclusively with either Xience stents (*n* = 1000) or bare metal stents (BMSs) (*n* = 500), the adverse clinical event rates were low at the 12-month follow-up with a comparable major adverse cardiac event rate (MACE). The aim of an important study by Conradie et al. was to examine the impact of sex at 1-year post-PCI on unplanned cardiac rehospitalizations [7]. A large cohort of patients (*n* = 13,996) was assessed for unplanned cardiac rehospitalizations within the first year post-PCI, and those admitted were followed in year 2 (post-PCI) for their survival status. Overall, the rate of unplanned cardiac rehospitalizations was 10.1%, with women having a remarkably high risk of unplanned cardiac rehospitalization (HR 1.29, 95% CI 1.11 to 1.48; *p* = 0.001). Any unplanned cardiac rehospitalization in the first year post-PCI was strongly associated with an increased all-cause mortality rate (HR 2.50, 95% CI 1.67 to 3.75; *p* < 0.001). Finally, in the third study by Conradie et al., an attempt was made to assess health-related quality of life (HRQoL) at baseline and its association with outcomes in patients with CAD undergoing PCI [8]. The EQ-5D-5L and VAS patient survey were utilized for the assessment of baseline HRQoL. Of the 15,198 eligible patients, only 6591 (43.4%) completed the self-assessment, and all-cause mortality and MACE rates at 1 year were obtained. The important findings of this study are that (a) women had significantly higher levels of impairment for all five dimensions of the EQ-5D-5L survey, and the self-reported QoL was significantly lower than in men (68.3 in women vs. 71.9 in men, *p* < 0.001); and (b) a poor QoL was strongly associated with increased all-cause mortality (HR 2.85; 95% CI 1.76 to 4.62, *p* < 0.001) and MACE rates (HR 1.40; 95% CI 1.10 to 1.79, *p* = 0.01). Both studies by Conradie et al. show important differences between men and women and provide insight into the importance of continuous surveillance of patient undergoing PCI.

This Special Issue includes several interesting papers that will increase the reader’s knowledge and understanding of the clinical frontiers in PCI. As a Guest Editor, I would like to thank all the contributing authors for their efforts, the reviewers for providing high-quality feedback, and the *JCM* editorial team for their support and assistance with this Special Issue.
